# The Expanding Role of Radiosurgery for Brain Metastases

**DOI:** 10.3390/medicines5030090

**Published:** 2018-08-14

**Authors:** Mark O’Beirn, Helen Benghiat, Sara Meade, Geoff Heyes, Vijay Sawlani, Anthony Kong, Andrew Hartley, Paul Sanghera

**Affiliations:** 1 Hall-Edwards Radiotherapy Research Group, Queen Elizabeth Hospital Birmingham, Edgbaston, Birmingham B15 2TH, UK; mark.obeirn@nhs.net (M.O.); helen.benghiat@uhb.nhs.uk (H.B.); sara.meade@uhb.nhs.uk (S.M.); geoff.heyes@uhb.nhs.uk (G.H.); anthony.kong@uhb.nhs.uk (A.K.); andrew.hartley@uhb.nhs.uk (A.H.); 2 Neuroradiology, Queen Elizabeth Hospital Birmingham, Edgbaston, Birmingham B15 2TH, UK; vijay.sawlani@uhb.nhs.uk

**Keywords:** stereotactic radiosurgery (SRS), stereotactic radiotherapy (SRT), brain metastasis, immunotherapy, whole brain radiotherapy (WBRT)

## Abstract

Stereotactic radiosurgery (SRS) has become increasingly important in the management of brain metastases due to improving systemic disease control and rising incidence. Initial trials demonstrated SRS with whole-brain radiotherapy (WBRT) improved local control rates compared with WBRT alone. Concerns with WBRT associated neurocognitive toxicity have contributed to a greater use of SRS alone, including for patients with multiple metastases and following surgical resection. Molecular information, targeted agents, and immunotherapy have also altered the landscape for the management of brain metastases. This review summarises current and emerging data on the role of SRS in the management of brain metastases.

## 1. Background

**Figure 1:**
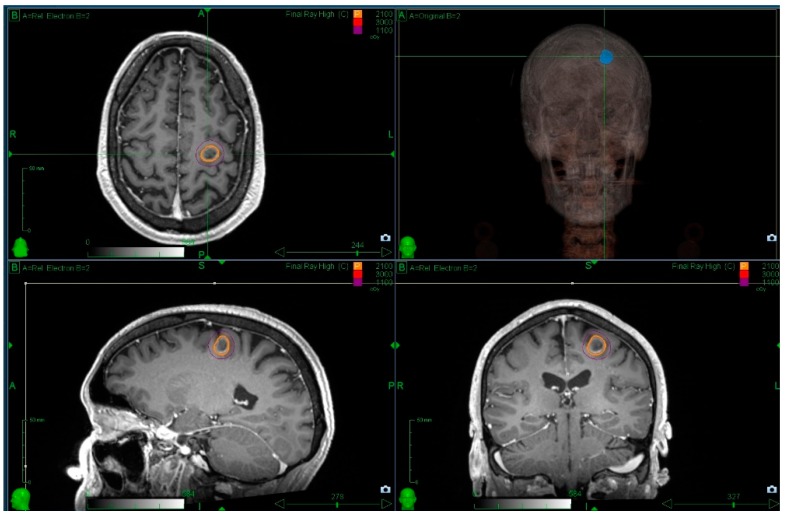
Radiosurgery for patients with a single metastasis and a favourable prognosis is standard practice. The figure illustrates a single metastasis covered with a prescription dose of 21 Gy prescribed to the 70% isodose line using Cyberknife. Prior informed consent was obtained for use of these images.

Intracranial stereotactic radiosurgery (SRS) was pioneered in the 1950s by the Swedish neurosurgeon Lars Leksell. The technique relies on multiple beams of radiation intersecting at a target precisely located within three dimensions. A single high dose of radiation is delivered with very rapid dose fall off in order to minimise the risk of damage to any adjacent tissue. The Gamma Knife was the first commercial intracranial SRS system and was based on Lars Leksell’s initial prototype, utilising fixed cobalt sources and surgical immobilisation of the skull to manage motion [1]. Subsequently the technique was replicated using linear accelerators subject to additional quality assurance and motion management strategies [2]. The Cyberknife is a specialist linear accelerator, mounted on a robot, which is capable of delivering SRS without the need for surgical immobilisation of the skull. Small well-demarcated tumours are the most suitable targets for SRS and brain metastases are now one of the most common indications (See Figure 1).

Brain metastases represent a significant cause of cancer morbidity, occurring in around 30% of patients with a malignancy originating outside the central nervous system [3]. The management of intracranial metastatic disease is made complicated by the impermeability of the blood-brain barrier to many chemotherapeutic agents, rendering this region a ‘sanctuary site’ for malignancies, most commonly breast, lung, melanoma, and renal cell carcinoma [4]. In 1999, a single-centre randomised controlled trial (RCT), in which patients with 2–4 brain metastases were treated with whole-brain radiotherapy (WBRT), alone or in combination with SRS, was stopped early due a significant improvement in the local control of the combination arm. All the patients had a local failure after 1 year within the WBRT group, compared to 8% following WBRT with SRS [5]. In 2004, the RTOG 9508 randomised trial demonstrated that the addition of SRS to WBRT improved survival for patients with a single metastasis compared with WBRT alone (median survival 6.5 months vs. 4.9 months, *p* = 0.04). Patients in the SRS group were more likely to have a stable Karnofsky Performance Status score at six months compared to WBRT alone (43% vs. 27% respectively, *p* = 0.03) [6]. These early studies helped to establish a role for SRS. As the principle benefit of SRS was durable control, several groups developed tools to help select those patients who were more likely to have a prolonged survival [7,8,9,10,11,12,13]. One of the earliest and simplest tools was the recursive partitioning analysis (RPA) derived from patients treated with WBRT within RTOG studies [14]. Patients were assigned to one of three prognostic groups based on performance status, age, presence of extracranial disease, and control of primary disease. Median survivals were 7.1, 4.2, and 2.3 months within RPA groups 1, 2, and 3, respectively. Although patients within group 1 would be candidates for SRS, the range within the middle category was broad. Subsequently, more sophisticated tools such as the diagnosis specific graded prognostic assessment (dsGPA) have been developed, which take into account the primary disease site to help selection [10]. However, an analysis evaluating the utility of six tools found that the benefits with more sophisticated tools were modest [15]. Recently the dsGPAs have been updated for selected primary sites, taking into account molecular information where possible [16,17].

Despite the modest survival benefit limited to a single metastasis within the original RTOG randomised trial [6], ongoing improvements in systemic survival, imaging, and more efficient SRS technology have led to a considerable expansion in practice. Furthermore, the potential for WBRT associated neurotoxicity in long-term survivors has led to a shift in practice [18]. The purpose of this review is to provide an update on the use of SRS for brain metastases within the contemporary era.

## 2. Methodology for Search Selection

Although not a systematic review, a literature search of Medline, the Cochrane Library, CINAHL, Embase, and Google Scholar was performed using the following terms: “Stereotactic radiosurgery”, “Stereotactic radiotherapy”, “SRS”, “SRT” and “Brain Metastasis”, “Immunotherapy”, or “resection”. The search focused on prospective studies, meta-analyses, systematic reviews, and retrospective studies with greater than 20 cases.

## 3. Discussion

### 3.1. Whole Brain Radiotherapy in Combination with Radiosurgery

WBRT was the historic standard of care prior to the widespread use of SRS [19]. However, the principle concern with WBRT, particularly in patients with a more favourable prognosis, is the negative impact on neurocognitive function (NCF) and quality of life [20]. NCF data is challenging to collect beyond a year, and baseline impairment often occurs in the presence of brain metastases [21,22,23]. An imaging study with a median follow-up of 6.25 years post-WBRT did show a significant impact on the rate of cerebral atrophy [24], and several clinical studies have reported shorter term outcomes comparing WBRT with observation following SRS.

Aoyama et al. assessed 132 patients randomised to SRS alone versus SRS with WBRT; they reported no differences in overall survival or NCF using a mini mental state examination (MMSE) [25]. However, this study was stopped early due to the sample size required to detect a survival difference, and the MMSE is a poor tool to evaluate post WBRT NCF impairment [26]. Chang et al. used a validated NCF tool and randomised 58 patients with 1–3 metastases between SRS alone and SRS with WBRT. The study was stopped early based on an interim analysis of 31 patients. Patients assigned to the WBRT group were more likely to have deterioration in the primary endpoint of total recall at 4 months, as measured by the ‘Hopkins Verbal Learning Test-Revised’ [27]. The mean posterior probability of decline was 52% in the SRS plus WBRT group versus 24% in the SRS alone group. A larger multi-institutional study randomised 213 patients between SRS alone and SRS plus WBRT with a primary endpoint of cognitive deterioration using a validated battery of tests. There was also significantly greater deterioration at 3 months using SRS with WBRT compared to SRS alone, 91.7% versus 63.5% (difference, −28.2%, 95% confidence interval (CI), −41.9% to −14.2%, *p* < 0.001). The quality of life also improved with SRS alone at 3 months (*p* = 0.001). The median follow-up was only 7.2 months, however, within a small subset of longer term survivors selected cognitive benefits were seen at 12 months with cognitive deterioration in executive function occurring more frequently after SRS plus WBRT compared with SRS alone, (42.9% vs. 0.0%, respectively; difference, 42.9%, 95% CI, 7.8%–77.9%, *p*  = 0.05) [28]. A multi-centre European study randomised 359 patients with 1–3 metastases undergoing SRS or surgery between observation and adjuvant WBRT. There was no difference in the primary endpoint of duration of patients’ functional independence (*p* = 0.89) [29]. A secondary endpoint was quality of life and patients had significantly higher global health related quality of life mean scores at 9 months with observation alone [30].

These aforementioned RCTs have consistently shown that WBRT improves intracranial control of disease, predominantly through a reduction of distant brain relapse, without a survival advantage [25,27,28,29]. A meta-analysis of individual patient data within the 3 of these RCTs evaluated 364 out of 389 pooled patients. Subset analyses were performed, and patients with a single metastasis had a significantly lower risk of distant brain failure than patients with 2–4 metastases (HR = 0.63, CI = 0.56–1.14). Age was an important factor as a continuous variable, and there was no difference in distant brain relapse between observation and WBRT in patients aged ≤50 years. There was also a significant survival advantage for patients aged ≤50 years with SRS alone and hazard ratios for patients 35, 40, 45, and 50 years of age were 0.46 (95% CI, 0.24–0.90), 0.52 (95% CI, 0.29–0.92), 0.58 (95% CI, 0.35–0.95), and 0.64 (95% CI, 0.42–0.99), respectively [31]. The only data to support a survival advantage from the addition of WBRT to SRS comes from a post-hoc analysis of lung cancer patients treated with SRS alone or SRS plus WBRT within the JROSG 99-1 study [32]. Patients with a favourable prognosis of lung cancer (dsGPA 2.4–4.0) had a survival advantage, with a median survival of 16.7 months (95% CI, 7.5–72.9) versus 10.6 months (95% CI, 7.7–15.5) (*p* = 0.04). As with any observation strategy, the ability to detect progression and offer prompt salvage therapy is important to reduce the chance of neurological morbidity and death. However, most now regard the new standard for patients with 1–3 brain metastases as SRS alone [18].

### 3.2. Radiosurgery Following Surgical Resection

Surgical excision is preferred for brain metastases where histological confirmation is required. Alternatively, the removal of large symptomatic masses can improve quality of life and permit more prompt withdrawal of steroids. For these reasons, a direct comparison of SRS to surgical resection has proven difficult with one attempted prospective study failing to accrue and closing early without providing an answer [33].

Failure in the surgical bed occurs in approximately 60% of cases at 2 years following resection alone and this is significantly reduced by adjuvant WBRT [29]. However, due to WBRT associated neurotoxicity, there has been increasing interest in conformal therapy to the surgical cavity including SRS. In a single centre RCT, patients who had a complete resection of 1–3 brain metastases were randomly assigned to SRS to the cavity or observation. The 12-month freedom from local recurrence was 72% (95% CI, 60–87) in the SRS group compared with 43% (95% CI, 31–59) in the observation group, with no adverse events or treatment-related deaths [34]. The median time to recurrence was 7.6 months in the observation group (95% CI, 5.3 months to NR) and not reached in the SRS group (95% CI, 15.6 months to NR). There was no difference in median overall survival (OS). Metastasis size was inversely correlated with better local control (LC) and tumours below 2.5 cm had a 90% freedom from local recurrence. In a multicentre RCT, patients with a single resected brain metastasis were assigned to cavity SRS or WBRT with the co-primary endpoints of cognitive-deterioration-free survival and OS. Patients with a cavity of <5 cm were included, and the dose was determined by the surgical cavity volume. The target volume consisted of the surgical cavity plus a 2 mm margin. The cognitive deterioration-free survival was slightly longer in patients assigned to SRS compared with those assigned to WBRT: 3.7 months versus 3 months respectively. Cognitive deterioration at 6 months was more frequent in patients who received WBRT than with those who received SRS (85% versus 52% respectively. Difference −33.6% (95% CI, −45.3 to −21.8), *p* < 0.00031). There was no difference in the median OS between the groups, however the SRS group had inferior 6 month LC compared to WBRT: 80.4% versus 87.1% respectively (*p* = 0.00068). 12 month LC rates were also inferior (61% versus 81%) and there was a shorter time to intracranial tumour progression: median 6.4 months versus 27.5 months in the WBRT group (*p* < 0.0001) [35]. In a small Polish study, patients undergoing resection of a single brain metastasis were randomised to WBRT or SRS or hypofractionated stereotactic radiotherapy (hfSRT). A dose of 15–18 Gy was used, or 25 Gy in 5 fractions for cavities larger than 5 cm or irregular in shape. The study was underpowered to make conclusions, although the surgical bed relapse was similar in both arms, and distant brain relapse was reduced with WBRT. Unlike other trials, the two-year OS rates appeared better in the early WBRT group than in the SRS group: 37% versus 10%, *p* = 0.015 [36].

Although the LC with cavity SRS may be lower than with WBRT, the improvement over observation with minimal associated morbidity has led many to adopt this as standard practice. The suitability of SRS for treating targets of a larger size and irregular shape is challenging, due to the interpretation of postoperative changes. The approach requires cooperation with a neuro-radiologist familiar with SRS and is often suited to small cavities, many of which may be suited to primary SRS alone. Recent guidelines serve to maximise LC when choosing cavity SRS [37]. It is possible that fractionated conformal therapy offers the benefits of improve LC without WBRT associated neurotoxicity; however, this remains to be evaluated prospectively against SRS or observation.

### 3.3. The Role of Radiosurgery for Multiple Metastases

Due to the aforementioned reasons, the use of WBRT has declined for patients with a favourable prognosis. With advances in SRS technology, the ability to efficiently treat a larger number of metastases has evolved and has become routine practice in a number of centres, although high quality evidence does not yet exist to support this approach. RCTs are currently open and evaluating the role of SRS alone versus WBRT for patients with multiple (≥4) metastases with regards to quality of life and neuro-cognition primary endpoints. While awaiting these data to inform practice, we present a summary of the published clinical evidence.

Yamamoto et al. [38] present the largest observational series of patients treated with primary SRS alone and no previous WBRT. This paper demonstrated non-inferiority in OS between cohorts of patients treated with SRS for 2–4 as compared to 5–10 metastases (10.8 months in both groups, *p* = 0.78). LC and distant brain failure rates were also not significantly different between these cohorts; suggesting that the number of metastases does not influence the local control outcome or the likelihood of distant relapse in the brain. The risk of presenting with leptomeningeal disease however was significantly higher in those patients with the largest number of metastases (5–10). Notably, the median total volume of metastases was similar between the two cohorts with multiple metastases, illustrating the importance of the total volume of disease when selecting patients with more numerous metastases. Furthermore, these are often highly selected patients based on a favourable prognosis.

A number of retrospective series have also reported outcomes of patients treated with SRS for ≥4 brain metastases with OS data presented in Table 1 for selected series. Notably, all of these series contain a proportion of patients treated with SRS for multiple metastases who had already received WBRT [39,40,41,42]. It is currently unknown whether the use of SRS upfront versus post WBRT differs in outcome.

It appears that survival outcomes for patients with multiple lesions treated with SRS are not inferior to those with fewer lesions; however, as with all single-centre, retrospective data, this is subject to bias and prospective evaluation is required. Many confounding variables influence the outcome in patients with multiple brain metastases, most importantly, performance status, age, histology, and whether the primary tumour or systemic disease is controlled. As such, it is not possible to recommend a single therapeutic option of either SRS alone or with WBRT at present. Patients with multiple metastases should be managed by a multi-disciplinary team with access to SRS. Those treated with SRS alone should receive a regular surveillance MRI, as rates of distant brain failure are high and salvage therapy should be offered (whether further SRS or WBRT) prior to neurological deterioration.

In conclusion, it is likely that both SRS and WBRT are important therapies in the management of patients with multiple brain metastases. However, the timing and combination of these still remain debatable.

### 3.4. Hypofractionated Stereotactic Radiotherapy

For large lesions or lesions close to critical structures, the delivery of stereotactic radiotherapy in multiple fractions may be radiobiologically advantageous, due to the higher tolerances of normal tissues to fractionation. Table 2 details fifteen series employing 2–7 fractions of radiotherapy where 12 month local control rates ranging between 49% and 96% are reported [43,44,45,46,47,48,49,50,51,52,53,54,55,56,57]. Studies were excluded from Table 2 if 12-month LC outcomes were not analysed, or if hypofractionated stereotactic radiotherapy (hfSRT) was performed postoperatively to the tumour cavity, with these patients not being separated in the analysis [58,59,60,61,62,63,64].

The data are difficult to interpret, as detail on the size of lesions treated is omitted in some series, and the use and size of a radiotherapy planning target volume (PTV) margin is variable. In addition, some series included patients who had received WBRT. In three series, where less than 10% of patients received WBRT, the rate of 12-month local control in patients with tumours >2 cm control was 74–85% [45,53,57]. In two series, where significant numbers of patients received WBRT, the corresponding range was 38–80% [47,49]. A comparison of late toxicity rates between these series is hampered by heterogeneity in reporting and definition. However, the rates of reported significant late toxicity appear low.

A pooled quantitative analysis of 10 retrospective studies of hfSRT demonstrated higher rates of local control when higher biological effective dose regimes were used [65]. Although hfSRT is in widespread use, a definitive prospective study to define appropriate dose, fractionation, and provide the evidence base for this treatment is desirable. Further retrospective studies are likely to be confounded by the factors described above.

### 3.5. Combining Immunotherapy with SRS

The ability to boost the immune response against malignant tumours has been one of most significant oncological advances in recent years. New agents are used to target checkpoint pathways, including the cytotoxic T-lymphocyte–associated antigen 4 (CTLA-4) pathway, which downregulates early T-cell function, and the programmed death 1 (PD-1) pathway, which regulates T-cell activity. Immune checkpoint inhibition (ICI) prevents the downregulation of T-cell activation and potentiates the anti-tumour response. Prolonged survival from metastatic disease has been demonstrated in several cancers, including melanoma, non-small cell lung cancer, and renal cell cancer [66,67,68,69]. Early RCTs excluded patients with brain metastases, but recent phase II data support that ICI can have an impact within the brain [70,71].

There has been significant debate with regards to a synergy between ICI and radiotherapy, including SRS. The immune cells including both T cells and myeloid cells are critical in mediating treatment response to radiotherapy [72,73,74]. Radiotherapy has been shown to induce the accumulation of myeloid-derived suppressive cells (MDSCs) and upregulate programmed death ligand 1 (PD-L1) expression in the tumour microenvironment, restricting its anti-tumour effect [75]. The addition of PD-1 antibody with radiotherapy has been shown to be synergistic in inhibiting tumour growth, as well as mediating an abscopal effect on distant non-irradiated tumours, by increasing CD8+ T cell response and reducing the local accumulation of MDSCs through the Tumour Necrosis Factor (TNF) [75]. In addition to preclinical models, PD-L1 expression and tumour-infiltrating lymphocytes have been found in brain metastasis samples across several solid tumour sites supporting the hypothesis of benefit from targeting this pathway [76,77,78].

Although an early retrospective series evaluating a mixture of systemic therapies suggested an increased risk of toxicity with concurrent ICI [79], evidence is now mounting in favour of a combination approach. A single prospective phase I study has confirmed the short-term safety of the CTLA-4 antibody ipilimumab with SRS [80]. Several retrospective series have also reported the safety profile of SRS with ipilimumab to be similar to SRS alone [81,82,83,84,85,86]. Some studies have suggested outcomes were better with a combined approach of ipilimumab and SRS [83,86,87]. A combined institutional analysis of 99 patients with metastatic melanoma reported improved intracranial control using SRS within 5.5 months of ipilimumab (median 3.63 vs. 8.09 months, hazard ratio (HR) 2.07, 95% CI, 1.03–4.16, *p* = 0.041) [87]. In another study of SRS for melanoma patients with brain metastases, concurrent treatment with ipilimumab improved local control compared to sequential treatment of SRS after ipilimumab [86].

Prospective data of anti-PD1 therapy with SRS is lacking, but an analysis combining data from 2 prospective trials of patients with melanoma looked at 26 patients with brain metastases treated with SRS within 6 months of receiving nivolumab (either before, during, or after nivolumab administration). There was one grade 2 headache following SRS, but no other treatment-related neurological toxicities [88]. Several retrospective cohorts have reported acceptable toxicity and possible advantages in combining anti-PD1 therapy with SRS [89,90,91]. One retrospective study evaluated 623 brain metastases within 260 patients and used multivariate analysis (MVA) to explore the impact of sequencing and type of ICI [89]. Using MVA, OS for patients treated with SRS/SRT and concurrent ICI was significantly higher than with non-concurrent SRS/SRT (*p* = 0.006) and SRS/SRT alone (*p* = 0.002). OS was also better for patients receiving anti PD1 therapy compared to anti CTLA-4 therapy (*p* = 0.004). Concurrent ICI reduced the likelihood of new brain metastases (*p* = 0.045).

Phase III data have shown that combination CTLA-4 and PD-1 inhibition improves progression-free survival within melanoma establishing a new standard of care [67]. Phase II data have recently been reported to suggest that this combination approach also gives a greater response within the brain, leading some to question the need for radiotherapy [70]. However, the durability of local control with ICI therapy alone compared to SRS remains unclear. It is possible that combination ICI with SRS offers the optimum outcome with regards to intracranial control, and such a strategy is currently under evaluation for patients with limited melanoma brain metastases [92].

Available data to guide decisions using ICI with SRS are retrospective, subject to multiple confounding variables, and complicated by a dynamic landscape with regards to the licensing of new agents and combinations. It is important that SRS be incorporated into prospective studies and that patients with brain metastases are not automatically excluded from the evaluation of new agents.

### 3.6. Response Assessment Following Radiosurgery

Serial structural Magnetic Resonance Imaging (MRI) remains the standard method of assessing response following SRS to brain metastases. However, in assessing response to SRS, structural MRI is reliant upon contrast enhancement pattern changes and alterations in T2/fluid-attenuated inversion recovery (FLAIR) weighting, which cannot sufficiently differentiate local tumour recurrence from SRS-induced changes [93]. In a series of 500 brain metastases treated with SRS, Patel et al. reported that one-third of the lesions had a transient size increase following treatment, starting as early as six weeks, and could be observed up to 15 months post-SRS [94]. It is estimated that between 30–75% of radiographically enlarging SRS-treated brain metastases are due to radiation-related changes alone [95,96,97,98,99,100]. In recognition of the increasing proportion of patients with brain metastases treated with a more aggressive local treatment and the increasing inclusion of this patient cohort in clinical trials, the international multidisciplinary Response Assessment in Neuro-Oncology Brain Metastases (RANO-BM) working group has published a guideline on standard response and progression criteria based on MRI [93]. However, the guidelines acknowledge the challenges in response assessment following SRS, recommending a short interval follow-up MRI in situations of uncertainty and the consideration of more advanced tests dependent upon local experience.

The use of MRI with delayed contrast extravasation more than one hour following a contrast injection has been reported to show differentiation between tumour recurrence and radiation changes [101]. Zach et al. used delayed contrast to develop treatment response assessment maps for both primary and metastatic brain tumours. The study included 26 brain metastases evaluated with histological correlation and sensitivity/PPV for active tumour was reported as 100%/89%. Other advanced MRI methods that can monitor the physiological and metabolic properties of tumour are being investigated to help distinguish radiation change from progression. These include diffusion-weighted imaging (DWI), perfusion-weighted imaging (PWI), and MR spectroscopy (MRS). The authors have been investigating the use of apparent diffusion coefficient (ADC), relative cerebral blood volume (rCBV) and the relative metabolite levels, particularly the choline to creatinine ratio from MR spectroscopy (Cho:Cr). Although there is data supporting the utility of each of these methods [95,96,98,100], limitations remain such that no single method is currently validated. It is possible that a combination multiparametric MRI protocol may provide a higher degree of confidence in gauging brain metastasis response post-SRS; however, prospective validation is required.

### 3.7. Radiosurgery Technical Developments

**Figure 2:**
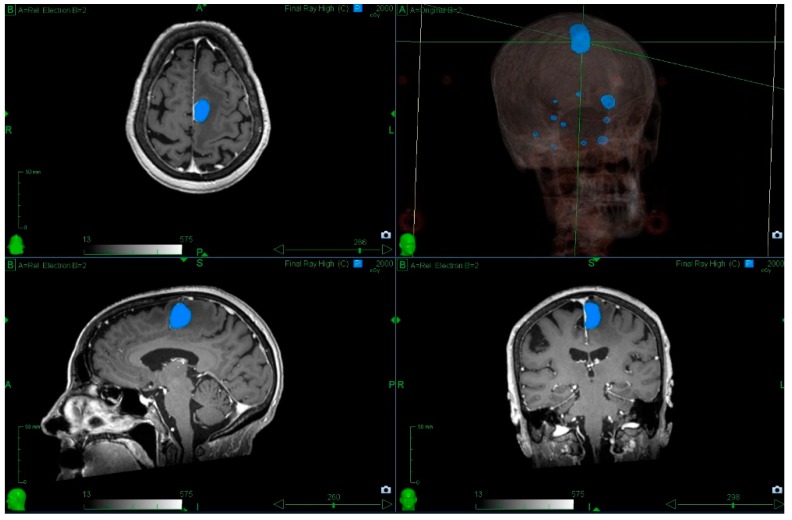
Radiosurgery to multiple metastases. The patient was referred with a symptomatic left frontal lobe metastasis; however, there were 11 metastases in total, and it was possible to treat all within a single session. The blue outline indicates the 20 Gy isodose (minimum dose prescribed to the left frontal lobe metastasis). Prior informed consent has been obtained for use of these images.

As discussed above, based on the available evidence, there is a trend away from WBRT and towards the use of SRS for multiple metastases. With modern dedicated SRS platforms, such as Gamma Knife and Cyberknife, it is possible to routinely treat multiple metastases in a single session (see Figure 2). Linear accelerators are more readily available than dedicated SRS machines and are also capable of delivering highly conformal therapy with increasing accuracy. Exploiting such readily available technology may help to meet the rising demand for SRS to brain metastases. Other advantages include the speed with which patients could be treated using techniques such as volumetric modulated arc therapy (VMAT) and the avoidance of surgical immobilisation. A summary of selected technical differences between SRS platforms is given in Table 3.

Conventional linear accelerator SRS uses several beams or arcs to treat individual targets at the isocentre and can prove inefficient when treating multiple metastases [102]. HyperarcTM (Varian Medical System Inc., Palo Alto, CA, USA) is a recent software development automating the delivery of multiple non-coplanar VMAT arcs to several targets around a single isocentre, thereby avoiding the need for verification at each isocentre [103,104]. HyperArcTM has been shown to be capable of delivering a higher Conformity Index, Gradient Index, and reduced treatment times compared to conventional multiple isocentre VMAT SRS [103]. At treatment planning level this single isocentre VMAT technique can achieve similar dose metrics to Gamma Knife and cyberknife for multiple metastases [102,104]. However, dedicated SRS machines such as Cyberknife and Gamma Knife routinely deliver treatment without the application of an uncertainty margin, including without surgical immobilisation, and this factor has to be taken into account.

Adapted conventional machines, with a comprehensive quality assurance programme, can achieve a 1-mm positional uncertainty for frameless deliveries with interfraction imaging [105]. For the treatment of multiple metastases, however, single isocentre treatments can lead to a magnification of spatial positioning uncertainties at increasing distances from the isocentre [106]. This leads to the requirement to add a CTV-PTV margin in order to ensure target coverage, which may be up to 2 mm for distant lesions [106]. Studies using non-invasive head frames and cone beam CT imaging have been shown to be able to achieve millimetre accuracy, but even with these systems, there is reluctance to treat metastases in eloquent locations [107]. Although there will be inherent uncertainties to all systems, the application of a margin must be considered carefully, given that it may be associated with an increased risk of toxicity [108]. Many of the published series to date have used platforms that have not applied a margin, establishing a reference standard of care. The combined steep gradient achievable via conical collimation and the ability to treat without a margin still makes these dedicated platforms highly suited for small multiple lesions.

## 4. Summary

SRS is being increasingly recognised as a valuable treatment option in the management of patients with brain metastases. Improvements in systemic disease control combined with recognition of WBRT toxicity have led to SRS becoming considered a standard of care in patients with 1–3 metastases. Surgical cavity SRS offers lower rates of neurocognitive deterioration at 6 months compared with postoperative WBRT, and despite inferior local control, survival rates are equivalent. Technical developments in planning have enabled treatments to be delivered to a greater number of metastases in a single session, whereas fractionating treatment may permit the treatment of larger volume lesions. Immunotherapy has changed the landscape for several cancers with a propensity to spread to the brain, and there may be a synergistic effect with SRS. Response assessment following SRS is complicated by post radiation change. Although advanced imaging techniques may aid its interpretation, it is difficult to recommend any single modality at present. Patients with brain metastases should be managed collaboratively by neuroscience centres having expertise in SRS, neurosurgery, and specialist imaging.

## Figures and Tables

**Table 1:** Retrospective series of patients with more than 4 metastases treated with SRS.

Reference	Year	Number of Metastases	Number of Patients	1 Year Rate of Distant Brain Failure	Median Overall Survival (Months)
Chang et al. [39]	2010	6–10	58	NR	10
		11–15	17	53.1%	13
		>15	33	80.3%	8
Mohammadi et al. [40]	2012	5–20	178	77.6%	4
Bhatnagar et al. [41]	2006	4–18	205	43%	8
Raldow et al. [42]	2013	5–9	84	NR	7.6
		≥10	19	NR	8.3

NR: not reported.

**Table 2:** Fractionated stereotactic radiotherapy for brain metastasis series.

Reference	Year	No. of Patients (Lesions)	Whole Brain RT	Median Dose/Fraction	Median GTV (cm^3^)	12-Month Local Control	Size Specific 12-Month Local Control
Aoyama et al. [43]	2003	87 (159)	0 (0%)	35 Gy/4#	3.3 (0.006–48.3)	81%	>3 cm^3^ 59%
Ernst-Stecken et al. [44]	2006	51 (72)	29 (57%) ^A^	30–35 Gy/5#	6 (0.29–65.57)	76%	NR
Aoki et al. [45]	2006	44 (65)	0 (0%)	24 Gy/4#	NR	72%	>2 cm diameter 79%
Narayana et al. [46]	2007	20 (20)	0 (0%)	30 Gy/5#	3.5 (2–5)	70%	NR
Giubilei et al. [47]	2009	30 (41)	30 (100%) ^A^	18 Gy/3#	4.8 (0.4–24.3)	86%	>2.1 cm diameter 80%
Higuchi et al. [48]	2009	43 (46)	0 (0%)	30 Gy/3#	17.6 (10–35.5)	76%	NR
Kwon et al. [49]	2009	27 (52)	NR ^B^	25 Gy/5#	NR	68%	>2 cm diameter 38%
Kim et al. [50]	2011	40 (49)	16 (40%)	36 Gy/6#	NR	69%	NR
Fokas et al. [51]	2012	61 (NR)	0 (0%)	35 Gy/7#	NR	75%	NR
		61 (NR)	0 (0%)	40 Gy/4#	NR	71%	NR
Märtens et al. [52]	2012	75 (108)	34 (45%) ^C^	35 Gy/7#	NR	52%	NR
		41 (52)	0 (0%)	35 Gy/7#	1 (0.1–19)	55%	NR
		34 (56)	34 (100%) ^C^	30 Gy/6#	2 (0.1–29.2)	49%	NR
Matsuyama et al. [53]	2013	299 (NR)	31 (10%) ^D^	36 Gy/2#	NR	95%	>2 cm diameter 85%
Rajakesari et al. [54]	2014	70 (NR)	40 (58%) ^E^	25 Gy/5#	NR	56%	NR
Minniti et al. [55]	2014	135 (171)	0 (0%)	27 Gy/3#	10.1 (1.6–48.4)	88%	NR
Navarria et al. [56]	2016	102 (102)	0 (0%)	27 Gy/3#, 32 Gy/4#	16.3 (3.9–64.5)	96%	NR
Marcrom et al. [57]	2017	72 (182)	5 (7%) ^F^	30 Gy/5#	2.02 (0.01–39)	86%	>3 cm diameter 61%, >2 cm 74%

RT: radiotherapy; GTV: gross tumour volume; Gy: Gray; #: number of fractions; NR: not reported; ^A^: WBRT followed by hfSRT boost; ^B^: 45/52 (86.5%) lesions treated with whole brain radiotherapy followed by hfSRT boost; ^C^: Median time from WBRT to hfSRT in months of 12.7 months (0.5–28.8); ^D^: Received WBRT before or after hfSRT; ^E^: 10 patients (14%) treated with hfSRT within 3 months of WBRT; ^F^: Previous WBRT, not given to lesions of interest.

**Table 3:** Technical differences between radiosurgery platforms.

**Platform Attributes**	**Modified Accelerator**	**CyberKnife**	**GammaKnife**
**Imaging Capability**	**Cone beam CT and Planar x-rays**	**Planar x-rays**	**Cone beam CT and optical monitoring**
Advantages	Inter-fraction compensation	Intra-fraction movement compensation with no patient repositioning	Intra-fraction monitoring when using relocatable headframe
	Ability to deliver prolonged fractionation	Hypofractionation	Hypofractionation
Disadvantages	Patient movement required to compensate intra-fraction shifts	Imaging dose during treatment	Intra-fraction imaging using optical surrogate. Patient movement required to compensate intra-fraction shifts
**Beam Delivery**	**Micro MLC**	**Cones, MLC**	**Cones**
Advantages	Homogenous dose distribution.	High dose conformality	High dose conformality.
	Speed of delivery		Lowest extracranial dose
Disadvantages	Reduced dose conformality	Inhomogeneous dose distribution.	Inhomogeneous dose distribution.
		Prolonged delivery	Prolonged delivery
**Geometric positioning**	**Gantry Mounted accelerator. Headframe**	**Robotic Mounted accelerator.**	**Static system.**
		**Immobilisation shell**	**Relocatable headframe**
Advantages	Arc therapy	Geometric precision < 0.5 mm	Geometric precision < 0.5 mm
		No PTV margin	No PTV margin
Disadvantages	Geometric uncertainty typically 1 mm	Limited ability to deliver posterior beams	Headframe.
			Delivery restricted to intra-cranial targets

CT: Computed Tomography; MLC: Multi-leaf collimator; PTV: Planning Target Volume.
